# Association between cardiometabolic index and liver function parameters: Analysis of NHANES 2007 to 2018

**DOI:** 10.1097/MD.0000000000048206

**Published:** 2026-04-10

**Authors:** Liqi Peng, Menglong Zou, Ruihua Huang, Ziming Peng

**Affiliations:** a Affiliated Hospital of Nanjing University of Chinese Medicine, Nanjing, China; b The First Clinical Medical College of Nanjing University of Chinese Medicine, Nanjing, China; c Department of Gastroenterology, Shenzhen Traditional Chinese Medicine Hospital, Shenzhen, China; d Fangchenggang Hospital of Chinese Medicine, Fangchenggang, China.

**Keywords:** alanine aminotransferase, aspartate aminotransferase, cardiometabolic index, liver function, metabolic diseases, NHANES

## Abstract

The cardiometabolic index (CMI) is widely used for risk prediction of metabolic diseases. This study examined the association between CMI levels and liver function parameters. This cross-sectional study analyzed data from the National Health and Nutrition Examination Survey from 2007 to 2018. The analysis encompassed 5 parameters of liver function: alanine aminotransferase (ALT), aspartate aminotransferase (AST), gamma-glutamyltransferase, alkaline phosphatase, and ALT/AST ratio. Multivariate weighted linear regression models were used to examine the relationships between CMI values, CMI quartiles, and liver function parameters, respectively. Subgroup analyses and interaction tests were conducted to account for potential confounding factors. The final sample comprised 9960 participants. Multivariate linear regression modeling demonstrated a significant positive association between CMI levels and ALT (*β* = 0.30, 95% CI = 0.20–0.41), AST (*β* = 0.19, 95% CI = 0.06–0.32), alkaline phosphatase (*β* = 0.17, 95% CI = 0.05–0.29), gamma-glutamyltransferase (*β* = 0.84, 95% CI = 0.29–1.40), and ALT/AST ratio (*β* = 0.01, 95% CI = 0.00–0.01). Positive correlations were observed between CMI quartiles and ALT, as well as the ALT/AST ratio. Subgroup analyses further demonstrated that the correlation between CMI levels and ALT and AST remained consistent across age, gender, and body mass index subgroups. CMI is positively associated with markers of liver dysfunction in US adults. Liver function screening in populations with elevated CMI levels should be prioritized to facilitate early intervention for liver disease.

## 1. Introduction

The liver is a vital organ that performs essential functions, including secretion, excretion, biotransformation, and immune regulation.^[1,2]^ Liver disease is a recognized global health problem that has garnered widespread recognition. The prevalence of nonalcoholic fatty liver disease (NAFLD) has been increasing annually in parallel with improvements in living standards.^[3,4]^ Globally, approximately 2 million people die each year from cirrhosis, liver failure, and liver cancer.^[5]^ The progression of liver disease is insidious, and abnormalities in liver function markers may appear before the development of portal hypertension or related complications. Alanine aminotransferase (ALT), aspartate aminotransferase (AST), alkaline phosphatase (ALP), gamma-glutamyltranspeptidase (GGT), and the ALT/AST ratio are the most widely used biomarkers for measuring liver function. Of these, ALT and AST are present in significant quantities within hepatocytes and serve as reliable indicators of parenchymal liver damage.^[6]^ Elevated levels of ALP and GGT are characteristic early manifestations of cholestasis.

The relationship between serum levels of liver enzymes and cardiovascular diseases (CVDs) has garnered significant research interest. A substantial body of research has demonstrated a positive correlation between serum levels of liver enzymes and the risk of developing CVDs.^[7,8]^ Studies have shown that an elevated ALT/AST ratio predicts the prevalence and mortality of CVDs across diverse populations.^[9,10]^ The cardiometabolic index (CMI) is a composite lipid index that reflects the distribution and function of visceral adipose tissue.^[11]^ A substantial body of research has validated the efficacy of CMI in predicting the onset of various CVDs, including heart failure,^[12]^ hypertension,^[13]^ and myocardial infarction.^[14]^ A cross-sectional study revealed a positive correlation between a higher CMI and a higher prevalence of CVD.^[15]^ Furthermore, related studies have revealed an L-shaped nonlinear relationship between CMI and hypertension.^[16]^ CMI has been demonstrated to be a useful tool for evaluating the risk of diabetes and renal insufficiency.^[17,18]^ Moreover, recent studies have identified potential associations between CMI and metabolic dysfunction-associated fatty liver disease (MAFLD) and NAFLD.^[19–21]^

CMI integrates information on key metabolic dysregulations such as visceral adiposity, lipid metabolism, and insulin resistance, which contribute to hepatic fat accumulation, low-grade inflammation, and early hepatocyte injury.^[22]^ These interconnected mechanisms suggest a potential link between elevated CMI and impaired liver function. However, the relationship between CMI and specific liver function biomarkers remains to be elucidated.

In this context, we hypothesized that higher CMI levels are associated with a greater likelihood of abnormalities in liver function indicators, including ALT, AST, ALP, GGT, and the ALT/AST ratio. To the best of our knowledge, this is the first cross-sectional study to investigate the association between CMI and liver function tests using data from a nationally representative U.S. population.

Accordingly, this study utilized National Health and Nutrition Examination Survey (NHANES) data from 2007 to 2018 to examine the association between CMI and multiple liver function biomarkers. Our findings provide novel insights and methodological support for the early screening and risk assessment of liver diseases.

## 2. Methods

### 2.1. Study population

**Figure 1.**
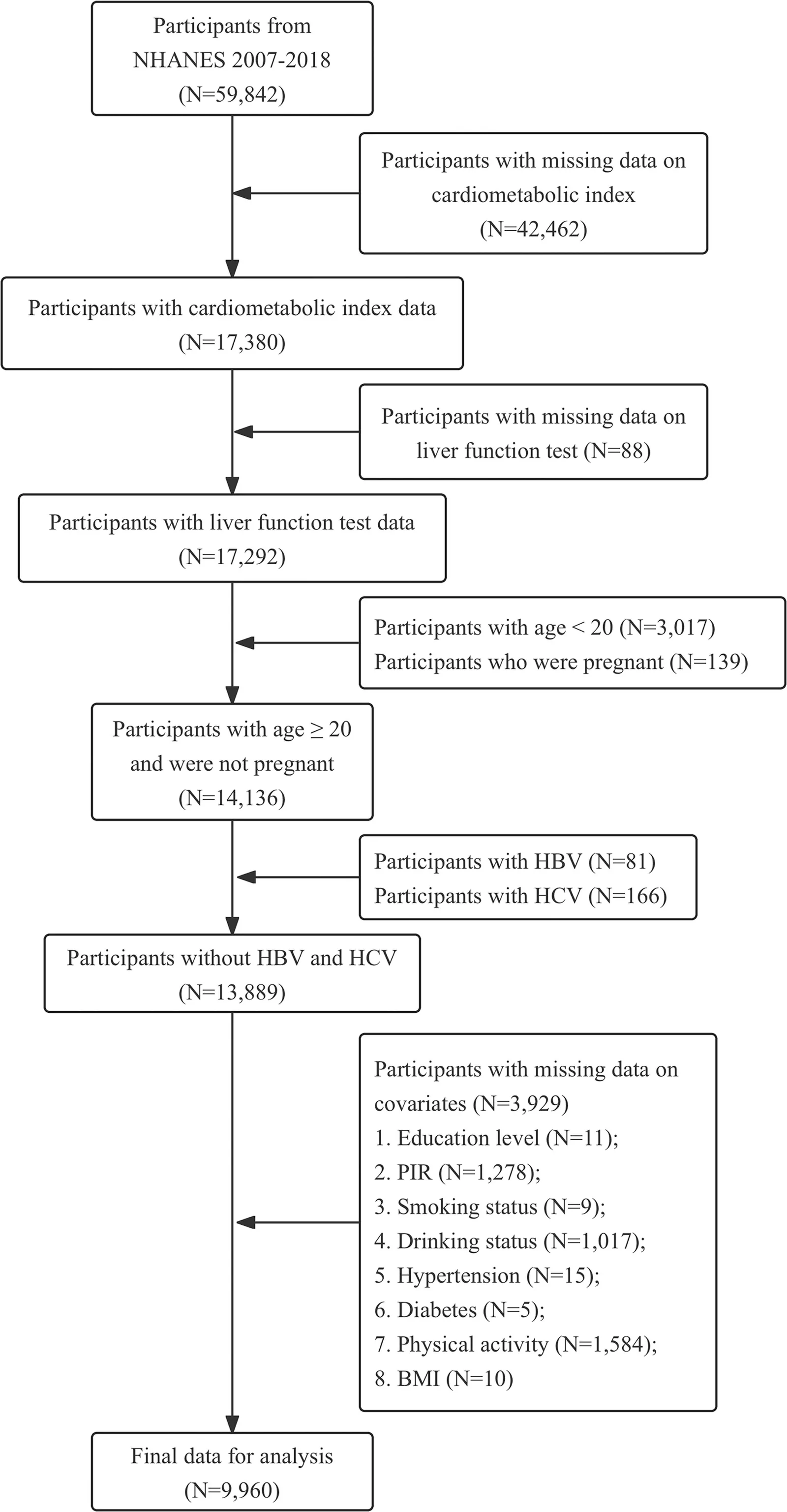
Flowchart of the screening process for study participants. BMI = body mass index; HBV = hepatitis B virus; HCV = hepatitis C virus; NHANES = National Health and Nutrition Examination Survey; PIR = poverty index ratio.

The NHANES includes participants of all ages, sexes, races, and geographic regions and collects data on demographics, nutritional intake, anthropometric measurements, biochemical indicators, and questionnaires.^[23–25]^ The data for this study were obtained from publicly available databases, and all participants in the NHANES study provided informed consent, so no additional ethical approval was required. Initially, 59,842 participants from the 2007 to 2018 NHANES cycles were included. Subsequently, participants lacking CMI or liver function data, those younger than 20 years, pregnant women, and participants with hepatitis B or C infection were sequentially excluded. Furthermore, 3929 participants were excluded due to missing data on covariates. The final analysis involved a total of 9960 participants (Fig. 1).

### 2.2. Calculation of CMI

The CMI was calculated as previously described in a prior study.^[12]^ The index was derived from measurements of triglyceride, high-density lipoprotein cholesterol, height, and waist circumference. The CMI was calculated using the following formula: CMI = [triglyceride (mmol/L)/high-density lipoprotein cholesterol (mmol/L)] × waist-height ratio. Waist-height ratio = waist circumference (cm)/height (cm). In accordance with prior epidemiological studies examining CMI and cardiometabolic risk,^[26]^ we categorized CMI into quartiles. Quartile categorization is a commonly used data-driven approach that allows for balanced group sizes and facilitates the assessment of potential dose–response relationships in large population-based datasets such as NHANES. In this study, the continuous CMI variable was categorized into quartiles: Q1 (CMI < 2.10), Q2 (CMI 2.10–3.29), Q3 (CMI 3.29–5.35), and Q4 (CMI > 5.35). Although alternative categorizations (e.g., tertiles or quintiles) were not used in the primary analysis, CMI was also analyzed as a continuous variable to assess the robustness of the results. The associations observed were consistent, indicating that the findings were not sensitive to the categorization method. Future studies with different population samples may explore alternative categorizations to further confirm these findings.

### 2.3. Liver function parameters

In this study, fasting blood samples were collected from participants aged 20 years and older. The samples were then stored at −30 °C until analysis. The liver function parameters described above were analyzed using the Beckman Synchron LX 20 (Beckman Coulter, Inc.‌, Brea) (enzymatic rate method) or the Beckman UniCel® DxC 800 Synchron (Beckman Coulter, Inc., Brea) (kinetic rate method).

### 2.4. Covariates

Based on prior literature and biological plausibility,^[24,27]^ we included a range of covariates that may confound the association between CMI and liver function. These covariates comprised demographic factors (age, sex, race, education level, and poverty impact ratio), lifestyle factors (smoking status, alcohol status, and physical activity [PA]), anthropometric measurement (body mass index), and health conditions (hypertension and diabetes). These variables were selected because they are known to influence both cardiometabolic status and liver function biomarkers, thereby potentially affecting the observed associations. In the description of participant characteristics and subgroup analyses, age was categorized as “≤60 years” and “>60 years.” Smoking status was determined by the inquiry, “Have you smoked at least 100 cigarettes in your entire life?” Drinking status was gauged through the inquiry, “Have you had at least 12 drinks of any type of alcoholic beverage?” The level of PA was stratified into 2 categories: “inactive” (<10 minutes per week) and “active” (≥10 minutes per week).^[28]^ Health status included the prevalence of hypertension and diabetes. Hypertension and diabetes were identified based on questionnaire data.

### 2.5. Statistical analysis

The statistical analysis was conducted using R software (version: 4.2.0; R Foundation for Statistical Computing, Vienna, Austria) and EmpowerStats software (version: 4.2; X&Y Solutions, Inc., Boston). Continuous variables were expressed using mean ± standard deviation, and comparisons between groups were made using the Kruskal–Wallis rank sum test. Categorical variables were expressed as counts and percentages, and comparisons between groups were performed using Fisher exact test. Multivariate weighted linear regression models were used to explore the association between CMI values (or CMI quartiles) and liver function parameters. Three models were employed for multivariate analyses. The crude model did not include any covariates. Model 1 was adjusted for 5 conventional variables: education level, age, gender, race, and poverty impact ratio. Model 2 was further adjusted for additional confounding variables, including smoking and drinking status, body mass index (BMI), PA, and health status. Furthermore, smoothed curve fitting was used to examine the relationship between CMI levels and liver function parameters. Subgroup analyses and interaction tests were used to assess the effect of covariates. A *P*-value <.05 was considered statistically significant.

## 3. Results

### 3.1. Baseline characteristics of participants

A total of 9960 participants were included in this study after screening. The mean CMI of the participants was 4.65 ± 5.83 (Table 1). The majority of participants were non-Hispanic White (46.56%), had attained at least a high school education (55.68%), and were classified as overweight (71.97%). Additionally, most participants reported engaging in <10 minutes of PA per week, and a substantial proportion were smokers and alcohol drinkers. The majority were non-hypertensive (62.44%) and did not have diabetes (84.16%). Significant differences in liver function parameters were observed across CMI quartiles (*P* < .05). The continuous variable CMI was then divided into quartiles. As CMI increased, all liver function parameters exhibited a gradual upward trend. These findings indicate a positive association between CMI and liver function parameters.

**Table 1:** Baseline characteristics of the participants in the NHANES study from 2007 to 2018.

Variables	Cardiometabolic index				*P*-value
	Q1 (<2.10)	Q2 (2.10–3.29)	Q3 (3.29–5.35)	Q4 (>5.35)	
*Demographic variables*					
Age, *N* (%)					<.001
≤60	1715 (68.88)	1646 (66.10)	1600 (64.26)	1769 (71.04)	
>60	775 (31.12)	844 (33.90)	890 (35.74)	721 (28.96)	
Sex, *N* (%)					<.001
Male	825 (33.13)	1080 (43.37)	1318 (52.93)	1612 (64.74)	
Female	1665 (66.87)	1410 (56.63)	1172 (47.07)	878 (35.26)	
Race, *N* (%)					<.001
Mexican American	238 (9.56)	356 (14.30)	385 (15.46)	443 (17.79)	
Other Hispanic	186 (7.47)	246 (9.88)	263 (10.56)	291 (11.69)	
Non-Hispanic White	1027 (41.24)	1149 (46.14)	1211 (48.63)	1250 (50.20)	
Non-Hispanic Black	787 (31.61)	514 (20.64)	370 (14.86)	239 (9.60)	
Other Race (including multiracial)	252 (10.12)	225 (9.04)	261 (10.48)	267 (10.72)	
Education level, *N* (%)					<.001
Less than high school	404 (16.22)	520 (20.88)	568 (22.81)	670 (26.91)	
High school or equivalent	503 (20.20)	560 (22.49)	621 (24.94)	568 (22.81)	
Above high school	1583 (63.57)	1410 (56.63)	1301 (52.25)	1252 (50.28)	
PIR, mean (SD)	2.72 (1.63)	2.57 (1.63)	2.59 (1.62)	2.38 (1.59)	<.001
*Lifestyle variables*					
BMI, *N* (%)					<.001
<18.5	50 (2.01)	50 (2.01)	35 (1.41)	13 (0.52)	
18.5–24.9	855 (34.34)	765 (30.72)	582 (23.37)	442 (17.75)	
≥25	1585 (63.65)	1675 (67.27)	1873 (75.22)	2035 (81.73)	
Physical activity, *N* (%)					.003
Inactive	1422 (57.11)	1464 (58.80)	1454 (58.39)	1346 (54.06)	
Active	1068 (42.89)	1026 (41.20)	1036 (41.61)	1144 (45.94)	
Smoking status, *N* (%)					<.001
Yes	901 (36.18)	1041 (41.81)	1180 (47.39)	1335 (53.61)	
No	1589 (63.82)	1449 (58.19)	1310 (52.61)	1155 (46.39)	
Drinking status, *N* (%)					<.001
Yes	1686 (67.71)	1684 (67.63)	1713 (68.80)	1815 (72.89)	
No	804 (32.29)	806 (32.37)	777 (31.20)	675 (27.11)	
*Health status variables*					
Hypertension, *N* (%)					<.001
Yes	830 (33.33)	880 (35.34)	994 (39.92)	1037 (41.65)	
No	1660 (66.67)	1610 (64.66)	1496 (60.08)	1453 (58.35)	
Diabetes, *N* (%)					<.001
Yes	208 (8.35)	291 (11.69)	391 (15.70)	451 (18.11)	
No	2220 (89.16)	2146 (86.18)	2038 (81.85)	1978 (79.44)	
Borderline	62 (2.49)	53 (2.13)	61 (2.45)	61 (2.45)	
*Biochemical variables*					
ALT (U/L), mean (SD)	20.78 (15.15)	22.66 (17.21)	25.29 (15.78)	29.60 (19.62)	<.001
AST (U/L), mean (SD)	24.15 (22.89)	24.14 (14.51)	25.11 (19.23)	26.66 (21.01)	<.001
ALP (U/L), mean (SD)	66.16 (22.64)	68.97 (23.73)	70.87 (26.58)	73.30 (24.64)	<.001
GGT (U/L), mean (SD)	23.90 (38.65)	25.38 (27.54)	28.80 (30.93)	37.24 (47.26)	<.001
ALT/AST, mean (SD)	0.87 (0.24)	0.93 (0.27)	1.01 (0.31)	1.10 (0.36)	<.001

ALT = alanine aminotransferase, AST = aspartate aminotransferase, BMI = body mass index, NHANES = National Health and Nutrition Examination Survey, PIR = poverty impact ratio, SD = standard deviation.

### 3.2. Relationship between CMI and liver function parameters

**Figure 2.**
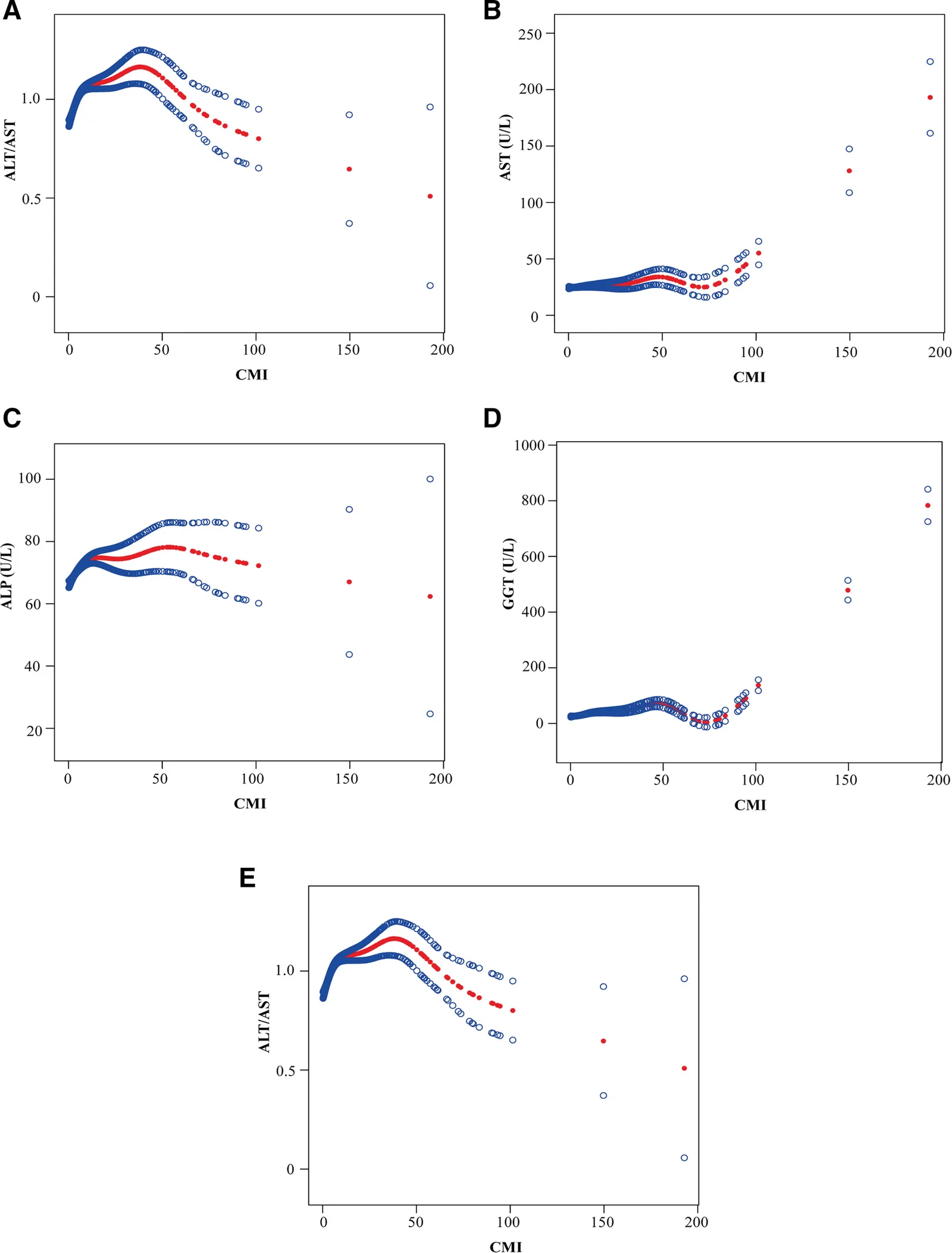
Smooth curves between CMI values and liver function parameters. (A) CMI and ALT; (B) CMI and AST; (C) CMI and ALP; (D) CMI and GGT; (E) CMI and ALT/AST ratio. ALP = alkaline phosphatase; ALT = alanine aminotransferase; AST = aspartate aminotransferase; CMI = cardiometabolic index; GGT = gamma-glutamyltransferase.

The associations between CMI values, CMI quartiles, and liver function parameters are presented in Tables 2 and 3. In the crude model, CMI values were positively associated with liver function parameters (*P* < .05). Each unit increase in CMI values resulted in a 0.51 U/L increase in ALT (*β* = 0.51, 95% CI = 0.41–0.61). Similarly, a one-unit increase in CMI values was associated with a mean increase of 0.25 U/L in AST levels (*β* = 0.25, 95% CI = 0.14–0.37). In addition, each one-unit increase in CMI was associated with a mean increase of 0.28 U/L in ALP (*β* = 0.28, 95% CI = 0.16–0.40), 1.07 U/L in GGT (*β* = 1.07, 95% CI = 0.56–1.58), and 0.01 in the ALT/AST ratio (*β* = 0.01, 95% CI = 0.01–0.01). These findings indicate that higher CMI values are associated with alterations in liver function parameters. These associations remained statistically significant in the fully adjusted Model 2 (*P* < .05). A positive correlation was identified between CMI values, CMI quartiles, and ALT and ALT/AST ratios, irrespective of model adjustments (*P* < .05). The impact of CMI level on liver function parameters was further substantiated by smooth curve fitting (Fig. 2).

**Table 2:** Results of multiple linear regression of the relationship between CMI values and liver function parameters.

Exposure	Outcome	*β* (95% CI), *P*-value		
		Crude	Model 1	Model 2
CMI value	ALT	0.51 (0.41–0.61) <.001	0.37 (0.27–0.48) <.001	0.30 (0.20–0.41) <.001
	AST	0.25 (0.14–0.37) <.001	0.21 (0.08–0.34) .002	0.19 (0.06–0.32) .006
	ALP	0.28 (0.16–0.40) <.001	0.26 (0.13–0.38) <.001	0.17 (0.05–0.29) .005
	GGT	1.07 (0.56–1.58) <.001	0.97 (0.44–1.51) <.001	0.84 (0.29–1.40) .004
	ALT/AST	0.01 (0.01–0.01) <.001	0.01 (0.00–0.01) <.001	0.01 (0.00–0.01) <.001

ALT = alanine aminotransferase, AST = aspartate aminotransferase, CMI = cardiometabolic index, GGT = gamma-glutamyltransferase.

**Table 3:** Results of multiple linear regression of the relationship between CMI quartiles and liver function parameters.

Exposure	Outcome	*β* (95% CI), *P*-value		
		Crude	Model 1	Model 2
*CMI quartile*				
Q1	ALT	Ref.		
Q2		2.11 (1.21–3.01) <.001	1.31 (0.41–2.21) .005	0.98 (0.07–1.89) .038
Q3		4.49 (3.57–5.41) <.001	3.01 (2.02–3.99) <.001	2.26 (1.25–3.28) < .001
Q4		8.78 (7.73–9.83) <.001	6.30 (5.16–7.45) <.001	5.06 (3.91–6.20) <.001
Q1	AST	Ref.		
Q2		0.05 (‐0.74–0.84) .910	‐0.23 (‐1.07–0.61) .589	‐0.34 (‐1.17–0.49) .423
Q3		0.75 (‐0.22–1.71) .133	0.18 (‐0.92–1.28) .752	‐0.06 (‐1.14–1.02) .916
Q4		2.57 (1.39–3.76) <.001	1.63 (0.25–3.02) .023	1.24 (‐0.07–2.55) .067
Q1	ALP	Ref.		
Q2		2.23 (0.77–3.69) .004	1.79 (0.31–3.26) .020	1.21 (‐0.28–2.69) .115
Q3		4.46 (2.52–6.41) <.001	4.03 (2.12–5.95) <.001	2.97 (1.09–4.86) .003
Q4		6.78 (5.12–8.43) <.001	6.26 (4.42–8.10) <.001	4.66 (2.83–6.50) <.001
Q1	GGT	Ref.		
Q2		1.70 (‐0.18–3.58) .080	1.02 (‐0.90–2.94) .303	0.33 (‐1.60–2.26) .741
Q3		5.56 (3.39–7.72) <.001	4.39 (2.10–6.68) <.001	2.84 (0.57–5.11) .017
Q4		12.27 (9.97–14.57) <.001	10.29 (7.85–12.73) <.001	7.78 (5.42–10.14) <.001
Q1	ALT/AST	Ref.		
Q2		0.07 (0.05–0.09) <.001	0.05 (0.03–0.07) <.001	0.04 (0.02–0.06) <.001
Q3		0.15 (0.13–0.17) <.001	0.11 (0.09–0.13) <.001	0.09 (0.07–0.11) <.001
Q4		0.24 (0.21–0.26) <.001	0.18 (0.16–0.20) <.001	0.14 (0.12–0.16) <.001

ALT = alanine aminotransferase, AST = aspartate aminotransferase, CMI = cardiometabolic index, GGT = gamma-glutamyltransferase.

### 3.3. Subgroup analysis

**Figure 3.**
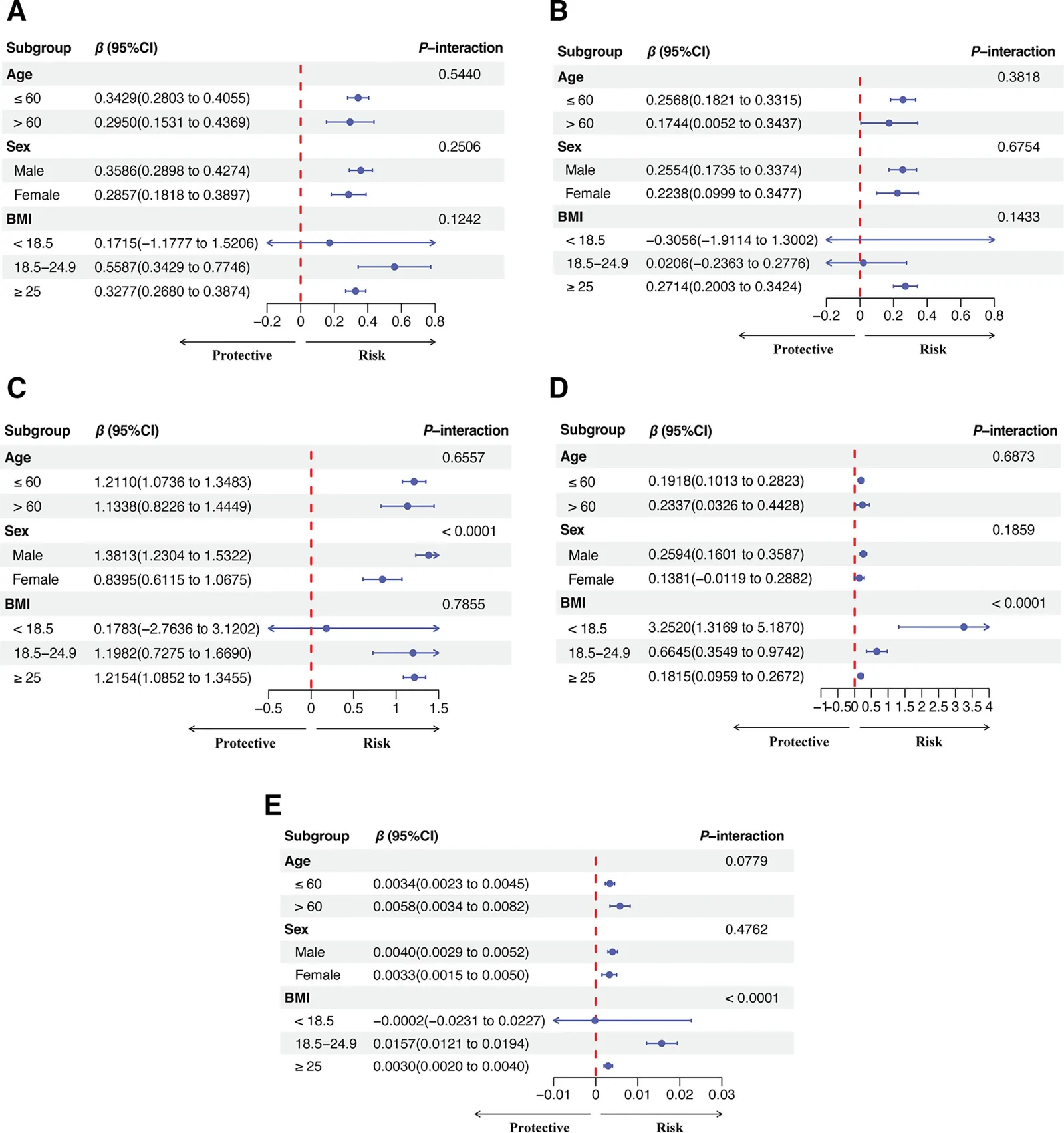
Subgroup analysis of CMI and liver function parameters. (A) CMI and ALT; (B) CMI and AST; (C) CMI and ALP; (D) CMI and GGT; (E) CMI and ALT/AST ratio. ALP = alkaline phosphatase, ALT = alanine aminotransferase, AST = aspartate aminotransferase, CMI = cardiometabolic index, GGT = gamma-glutamyltransferase.

Subsequently, subgroup analyses stratified by age, sex, and BMI were performed to assess the association between CMI levels and liver function parameters (Fig. 3). Interaction tests indicated no statistically significant differences in the associations between CMI and AST or ALT across age, sex, or BMI strata. These results indicate no significant interaction effects of age, sex, or BMI on the associations between CMI and liver function parameters (*P* > .05). However, significant associations were observed between higher CMI levels and increased ALP in sex-stratified analyses, GGT in BMI-stratified analyses, and the ALT/AST ratio in BMI-stratified analyses (*P* < .05).

## 4. Discussion

This study demonstrated a consistent positive correlation between CMI and liver function parameters. Elevated serum CMI levels were associated with markers of impaired liver function. Subgroup analyses further indicated that the associations between CMI and ALT and AST were consistent across gender, age, and BMI subgroups. These findings suggest that a lower cardiometabolic burden may be associated with more favorable liver function profiles.

CMI integrates 4 commonly used variables, is simple to calculate, and reflects the combined impact of waist-to-height ratio and lipid levels on metabolic diseases.^[29]^ CMI has broad clinical applicability and has been widely used in studies across diverse populations to assess cardiometabolic status and predict the risk of CVDs, diabetes, hyperuricemia, and other metabolic disorders.^[30,31]^ A related study noted a significant positive correlation between CMI levels and hepatic steatosis.^[32]^ This relationship between CMI and liver disease has been further validated in several population-based cross-sectional studies.^[20,21]^ The findings indicated that after excluding potential confounding factors, elevated CMI levels were significantly associated with an increased risk of NAFLD and MAFLD.^[33–35]^ Moreover, the study demonstrated that CMI showed greater diagnostic value for MAFLD in females than in males.^[19]^ These findings suggest that CMI may be a useful indicator associated with liver function parameters and may have potential value in identifying individuals with liver impairment.

The potential mechanisms linking elevated CMI to impaired liver function primarily involve lipotoxicity and insulin resistance. Excess visceral adiposity, particularly in the context of adipose tissue dysfunction, drives an increased flux of free fatty acids to the liver, promoting ectopic lipid accumulation, lipotoxic stress, mitochondrial dysfunction, and oxidative stress.^[36]^ These processes collectively induce hepatocyte injury and result in elevated aminotransferase levels. Adipose tissue dysfunction and visceral fat accumulation have been causally linked to hepatic insulin resistance and NAFLD severity.^[37,38]^ Additionally, insulin resistance, a key component of the metabolic dysregulation associated with higher CMI, further aggravates liver dysfunction. Insulin-resistant adipose tissue releases pro-inflammatory cytokines and free fatty acids, which in turn exacerbate hepatic insulin resistance, stimulate de novo lipogenesis, and promote the formation of toxic lipid species, such as ceramides.^[39]^ These events trigger endoplasmic reticulum stress, oxidative stress, and inflammatory signaling within hepatocytes, thereby driving liver injury and dysfunction. Moreover, this lipotoxic-inflammatory cycle is reinforced by recent evidence demonstrating that lipotoxic and insulin-resistant states interact via shared molecular mediators to amplify hepatic metabolic stress.^[40]^ These mechanisms provide a plausible biological basis for the observed positive association between elevated CMI and liver function parameters in our study.

The main biomarkers of liver function commonly used in clinical practice are ALT, AST, ALP, and GGT. ALT is an intracellular enzyme that catalyzes amino transfer reactions and exhibits its highest activity in the liver.^[41]^ AST is involved in intermediary metabolism and liver regeneration.^[42]^ Serum levels of both enzymes are widely recognized as specific indicators of liver dysfunction. In the event of hepatocyte damage or destruction, the release of ALT and AST into the bloodstream occurs, resulting in elevated levels of these enzymes. The extent of elevation in ALT is often more substantial than that of AST in the initial stages of liver disease. ALP catalyzes the hydrolysis of phosphate esters, producing phosphoric acid in alkaline environments. It plays an essential role in bile production, bone mineralization, and intestinal absorption.^[43]^ GGT catalyzes γ-glutamyl group transfer, participates in glutathione metabolism, and plays a key role in maintaining antioxidant homeostasis.^[44]^ Elevated levels of serum ALP and GGT have been observed in cases of cirrhosis, cholelithiasis, and tumor-induced cholestasis.^[45]^

Multi-omics studies have shown that AST and ALT not only reflect the extent of hepatic necrosis but also play regulatory roles in systemic energy metabolism. These studies have demonstrated a robust correlation between these enzymes and myocardial metabolism activity, as well as the incidence of cardiovascular events.^[46]^ Concurrent studies have reported that elevated AST levels and ALT/AST ratios are independently associated with CVD outcomes.^[10,47]^ Furthermore, ALT levels have been identified as being associated with increased interventricular septal thickness and left ventricular mass in individuals with metabolic syndrome, as well as in overweight or obese populations with NAFLD. In recent years, ALP has garnered increasing attention as a potential therapeutic target in CVDs.^[48–50]^ Significant associations have been reported between serum ALP levels and atrial fibrillation, atherosclerosis, and ischemic heart disease.^[51–53]^ GGT has emerged as a novel marker associated with inflammation, myocardial infarction, stroke, and cardiac death. Elevated serum GGT levels exhibit a strong correlation with cardiovascular events.^[54,55]^

In the subgroup analyses, the positive correlations between CMI levels and ALT and AST were not affected by age, gender, or BMI stratification. The interaction tests demonstrated statistically significant differences in the association between CMI and ALP across gender strata, with a more pronounced positive correlation observed in male participants. This finding may be partly attributed to gender-related differences in serum ALP levels. Preliminary studies have reported that ALP levels were higher in males between the ages of 20 and 49 years, whereas serum ALP levels were higher in females between the ages of 50 and 79 years.^[56,57]^ Furthermore, the associations between CMI and GGT, as well as the ALT/AST ratio, varied significantly across BMI strata. However, these associations were not entirely consistent across BMI categories. The positive association between higher CMI and GGT levels was more pronounced among underweight and overweight/obese participants compared with those of normal weight, suggesting that maintaining a healthy body weight may be beneficial for cardiometabolic and liver health. Consistent with this observation, previous studies have shown that increased body adiposity, particularly abdominal adiposity, is associated with adverse liver function parameters in healthy adult women.^[58]^ This observation is consistent with the results of the current study. Conversely, underweight status appeared to attenuate the association between CMI and the ALT/AST ratio. A multitude of studies have indicated that BMI can influence hypertension and serum uric acid levels in diabetic patients by mediating the ALT/AST ratio.^[59,60]^ However, the specific effects of the relationship between BMI and ALT/AST ratio in cardiometabolism are not fully understood. Further clinical studies are warranted to clarify these relationships.

This study has several strengths. First, this study utilized up to 12 years of NHANES data, providing a large, nationally representative sample. Second, to our knowledge, this is the first cross-sectional study to examine the association between CMI and 5 liver function parameters. These findings provide additional evidence supporting the association between cardiometabolic status and liver dysfunction.

Nevertheless, several limitations should be acknowledged. First, as with other NHANES studies, this cross-sectional investigation cannot establish causality. Second, although we considered numerous potential confounders, residual confounding from unmeasured factors such as dietary habits, medications, and genetic predispositions may still exist. Third, although CMI is practical, it may not fully capture visceral fat distribution or liver fat content. In addition, liver function markers (ALT, AST, ALP, and GGT) reflect hepatocellular injury but do not provide detailed information on liver pathology or disease severity. Fourth, both CMI and liver function parameters were measured at a single time point, limiting the assessment of temporal changes. Finally, the exclusive focus on US adults restricts the generalizability of the results to other populations or geographic regions.

## 5. Conclusions

This study demonstrated a positive association between CMI levels and liver function parameters among U.S. adults using NHANES data from 2007 to 2018. These findings indicate that higher CMI levels are associated with elevated liver enzyme concentrations, suggesting that lower cardiometabolic burden may be linked to more favorable liver enzyme profiles. Further research is required to corroborate our findings and to investigate the underlying mechanisms of the “heart-liver axis.”

## Author contributions

**Conceptualization:** Liqi Peng, Menglong Zou.

**Data curation:** Liqi Peng, Menglong Zou.

**Investigation:** Ruihua Huang.

**Methodology:** Ziming Peng.

**Software:** Liqi Peng, Menglong Zou.

**Supervision:** Ziming Peng.

**Visualization:** Ruihua Huang.

**Writing – original draft:** Liqi Peng.

**Writing – review & editing:** Menglong Zou, Ziming Peng.
